# Meat Quality, Fatty Acid Content and NMR Metabolic Profile of Dorper Sheep Supplemented with Bypass Fats

**DOI:** 10.3390/foods10051133

**Published:** 2021-05-19

**Authors:** Atique Ahmed Behan, Muhammad Tayyab Akhtar, Teck Chwen Loh, Sharida Fakurazi, Ubedullah Kaka, Azira Muhamad, Anjas Asmara Samsudin

**Affiliations:** 1 Department of Animal Science, Faculty of Agriculture, Universiti Putra Malaysia, Serdang 43400, Malaysia; atiquebehan@sau.edu.pk (A.A.B.); tcloh@upm.edu.my (T.C.L.); 2 Department of Livestock Management, Sindh Agriculture University, Tandojam 70060, Pakistan; 3 Institute of Industrial Biotechnology, Government College University, Lahore 54000, Pakistan; tayyabakhtar@hotmail.com; 4 Laboratory of Natural Products, Institute of Bioscience, Universiti Putra Malaysia, Serdang 43400, Malaysia; 5 Department of Human Anatomy, Faculty of Medicine and Health Science, Universiti Putra Malaysia, Serdang 43400, Malaysia; sharida@upm.edu.my; 6 Department of Companion Animal Medicine &Surgery, Faculty of Veterinary Medicine, Universiti Putra Malaysia, Serdang 43400, Malaysia; dr_ubedkaka@upm.edu.my; 7 Malaysia Genome Institute, National Institutes of Biotechnology Malaysia (MOSTI), Jalan Bangi, Kajang 43000, Malaysia; aziramuhamad@nibm.my

**Keywords:** Dorper sheep, fatty acids, metabolomics, nuclear magnetic resonance (NMR) spectroscopy, rumen bypass fat

## Abstract

The supplementation of rumen bypass fat (RBF) has remained one of the preferred approaches used to decrease undesirable saturated fatty acids (FA) and increase beneficial unsaturated FA in the meat. This study was planned to evaluate the influences of rumen bypass fats on meat quality, fatty acid and metabolic profiles in male Dorper sheep (*n* = 36) with 24.66 ± 0.76 kg (mean ± standard error) initial body weight. Treatment comprised a basal diet (30:70 rice straw to concentrate) with no added RBF as a control (CON), basal diet with prilled fat (PF), basal diet with prilled fat plus lecithin (PFL) and basal diet with calcium soap of palm fatty acids (CaS). The findings revealed that cooking loss, drip loss and shear force in *longissimus dorsi* (LD) muscle were not affected by RBF supplementation, while meat pH was significantly higher in the CaS on aging day 1. However, the diet supplemented with prilled fat and lecithin modified the meat’s fatty acid profile significantly by increasing unsaturated fatty acids and decreasing saturated fats. The relative quantification of the major differentiating metabolites found in LD muscle of sheep showed that total cholesterol, esterified cholesterol, choline, glycerophosphocholine and glycerophospholipids were significantly lower in CaS and PFL diets, while glycerol and sphingomyelin were significantly higher in CaS and PFL diets. Most of the metabolites in the liver did not show any significant difference. Based on our results, the supplementation of protected fats did not have a negative influence on meat quality and the meat from Dorper sheep fed prilled fat with lecithin contained more healthy fatty acids compared to other diets.

## 1. Introduction

Red meat is considered a principal dietary source of protein and essential nutrients—including vitamins and minerals that are important for human health. However, a review by McAfee et al. [1] reported several studies stating that red meat consumption may escalate the risk of cardiovascular diseases and cancer in the colon. On the other hand, n-3 polyunsaturated fatty acids (PUFA) and conjugated linoleic acid (CLA) are widely recognized for their positive impact on human heart health, improving platelet aggregation, vasodilation and thrombotic tendency [2,3].

In the last few years, there has been increasing interest in enriching unsaturated fatty acids (FA) and reducing saturated FA levels in ruminant products. Supplementation of rumen bypass fat has been one of the methods used for this modification [4]. Previous studies [5,6] conducted on dietary lipids have revealed that the addition of unprotected fats in ruminant diets has negligible influence on FA composition due to the biohydrogenation of unsaturated FA by microbes in the rumen. On the other hand, supplementation of rumen bypass fats also protects unsaturated FA from rumen biohydrogenation. Therefore, FA are subsequently absorbed in the small intestine and their incorporation in the muscle and adipose tissues of ruminants is potentially increased [7].

In this context, many studies have been conducted on the influence of dietary fats and FA composition on several aspects of meat quality [8,9,10,11]. Rumen bypass fat has been widely evaluated in dairy animals [12,13,14,15] and, to some extent, beef cattle [16,17,18]; however, these studies were mainly focused on growth performance and rumen fermentation characteristics. Hence, there have been a limited number of studies on rumen bypass fat, especially on sheep for meat purposes. Moreover, these studies are highly variable and inconsistent—and their influence on meat quality is obscure.

Metabolomics is the study of metabolites which are the low molecular weight compounds in biological systems resulting from metabolic activity [19]. In food science, in particular, metabolomics is used to explore the major compounds that contribute to the physicochemical properties, sensory assessment and nutritional quality of food [20]. Several studies have been conducted on metabolites in cattle to predict physicochemical meat quality traits, including the influence of animal genetic background [21], feeding [22,23,24], muscle type [25], postmortem aging [26,27] and meat processing [28]. While many of these investigations were performed on the influence of bypass fats on the growth performance, carcass characteristics, meat quality and FA composition of muscles, no study has been conducted, to our knowledge, on muscle and liver metabolomes of the sheep supplemented with rumen bypass fat (RBF). Hence, the current study is aimed at characterizing the metabolomes of the *longissimus dorsi* muscle and liver tissue of sheep supplemented with rumen bypass fats using ^1^H NMR-metabolomics approach. The research was planned to evaluate the influences of bypass fats on quality, fatty acid profile and metabolites in meat samples from Dorper sheep.

## 2. Materials and Methods

### 2.1. Animals, Housing and Treatments

The study was conducted as approved by the Universiti Putra Malaysia Animal Care and Use Committee (IACUC) guidelines (Reference # R064/2016). Male Dorper sheep (*n* = 36) with an age of 18 months and having 24.66 ± 0.76 kg (mean ± SE) average initial body weight were supplemented with experimental diets for 90 days. The housing and all management were similar to those described in our previous experiment [29]. The trial was conducted using completely randomized design (CRD) and sheep were assigned into 4 treatment groups (*n* = 9). The rumen bypass fats (RBF) commercially available in the market were purchased from two different companies. The four isonitrogenous and isocaloric diets, formulated as per the recommendations of the National Research Council [30], were: (1) basal diet with no added RBF as a control (CON); (2) basal diet with prilled fat (PF); (3) basal diet with prilled fat plus lecithin (PFL); and (4) basal diet with calcium soap (calcium salts of palm FA) (CaS) on DM basis at 5% of the dry matter (DM). The sheep were fed based on their individual weight—3% body weight on DM basis.

### 2.2. Chemical Analysis

Chemical analysis of the experimental diets was performed as per the Association of Official Analytical Chemists (AOAC), as described by Adeyemi et al. [31]. Neutral detergent fibre (NDF) and acid detergent fibre (ADF) were determined according to the protocol of Van Soest et al. [32], as described by Adeyemi et al. [31]. Table 1 and Table 2 show the composition of fatty acids and the diets, respectively, used in this study.

### 2.3. Slaughtering and Tissue Sampling

All the experimental sheep were slaughtered by Halal standard slaughter procedure, Malaysian Standards MS 1500:2009 Department of Standards Malaysia [33]. Following evisceration, the liver samples were collected from the right lobe of the liver. *Longissimus dorsi* muscle was excised from the 6th–8th lumbar vertebra. All the tissue samples were snap-frozen in liquid nitrogen (LN_2_) and stored at −80 °C.

### 2.4. Aging of Meat

The aging of the meat was performed as described by Adeyemi et al. [31]. Each muscle (60 g) was dissected and divided into 3 parts on day 0. The 1st part (about 15 g) was crushed into a homogenous powder with a porcelain mortar and pestle in LN_2_ and stored at −80 °C for pH and FA analysis. The 2nd part (about 15 g) was stored in a polyethene pack in a chiller at 4 °C for drip loss on day 1 and 7. The third part (about 30 g) was kept for color, cooking and shear force analysis.

### 2.5. pH Determination of Muscles

The pH of crushed muscles was determined using a precalibrated portable pH meter (Mettler Toledo, AG 8603, Greifensee, Switzerland).

### 2.6. Determination of Meat Colour Coordinates

Instrumental colour was analyzed using a ColorFlex (Hunter Associates Laboratory, Reston, VA, USA). The samples were analyzed for redness (a*), yellowness (b*), lightness (L*) and hue angle (arctan, b*/a*), which describes the hue or colour of the meat and chroma or saturation index calculated as

$$\surd \left(a^{2}+b^{2}\right)$$

This defines the vividness or brightness of colour.

### 2.7. Determination of Water-Holding Capacity (WHC)

#### 2.7.1. Drip Loss

To evaluate drip loss, about 30 g of sample taken at 0, 1 and 7 days postmortem were trimmed and weighed (*W1*). Thereafter, samples were placed in individual sealed polyethylene plastic bags, vacuumed, and kept at 4 °C for 24 h. Samples were then reweighed (*W2*). Drip loss was calculated as the % weight change [34].

$$\mathrm{Drip}\;\mathrm{loss}\;\%=\frac{W1-W2}{W1}\times 100$$

where:

*W1* = Initial weight of the sample
*W2* = Weight after the storage period.

#### 2.7.2. Cooking Loss

The samples were transferred from −80 °C freezer into a chiller (4 °C) and kept overnight for thawing. Then, they were weighed separately (*W1*), placed in polyethylene bags and inserted in a hot water bath (80 °C) until the internal temperature reached 78 °C. The samples (along with the polyethylene bags) were cooled for 15 min under tap water. Thereafter, samples were taken out of the polyethylene bags, dried with paper towels and weighed (*W2*). Cooking loss was determined by weighing the samples before and after cooking [34], as described by Adeyemi et al. [31].

$$\mathrm{Cooking}\;\mathrm{loss}\;\%=\frac{W1-W2}{W1}\times 100$$

where:

*W1* = Initial weight of the sample
*W2* = Weight of cooked sample.

### 2.8. Texture Analysis

The texture analysis was determined as described by Adeyemi et al. [31]. The samples used for cooking loss were used to determine Warner–Bratzler Shear force (WBS). The WBS was determined using TA. HD Plus Texture Analyzer fitted with a Volodkevitch bite jaw (Stable Micro System, Surrey, UK).

### 2.9. Analysis of Fatty Acids

Analysis of FA of meat and liver tissues was performed as described by Behan et al. [29]. The tissues were extracted in methanol:chloroform (1:2, *v*/*v*) mixture. The FA were transmethylated using 0.66 N KOH into their fatty acid methyl esters (FAME) in methanol and 14% methanolic boron trifluoride (BF3). The internal standard was heneicosanoic acid. The FAME was separated in a gas chromatograph equipped with a flame ionization detector (Agilent 7890A—Agilent Technologies, Palo Alto, CA, USA). Conjugated linoleic acid standard mixture (O-5507 Sigma-Aldrich, Inc., St. Louis, MO, USA) and a reference standard (mix C4-C24 methyl esters; Sigma-Aldrich, Inc., St. Louis, MO, USA) were used to determine individual FA composition.

### 2.10. Extraction of Tissue Metabolites and Preparation for NMR Spectroscopy

The muscle and liver samples were extracted according to established protocol [35] as described in Supplementary Materials File S1. Frozen tissues were pulverized to a fine powder with a porcelain mortar and pestle using liquid nitrogen. Combined metabolites (polar and nonpolar) were extracted using methanol:chloroform. The polar fraction (extracted with methanol) was resuspended in 550 μL D_2_O (100 m*M* sodium phosphate buffer, pH 7.4) containing 0.5 m*M* sodium 3-(trimethylsilyl) proprionate-2,2,3,3-d4 (TMSP; 98% purity), purchased from Merck (Darmstadt, Germany) and used as a reference standard. Then, the mixture was vortexed for 10 sec and centrifuged at 12,000× *g* for 5 min at room temperature. Likewise, the nonpolar fraction (extracted with chloroform) was resuspended in 550 μL 2:1 mixture of chloroform-d (CDCl_3_; 99.8% purity) and methanol-d_4_ (CD_3_OD; 99.8% purity) containing 0.03 vol. % of tetramethylsilane (TMS; 99.8% purity), purchased from Merck (Darmstadt, Germany) and used as a reference standard. Then, the mixture was vortexed for 10 sec and centrifuged at 1000× *g* for 5 min at room temperature. The samples were then transferred into labelled 5 mm NMR tubes and subjected to NMR analysis.

### 2.11. NMR Measurement and Data Processing

Proton (^1^H) NMR spectroscopy was conducted on a 700 MHz Bruker Avance (Bruker-Blospin, GmbH, Rheinstetten, Germany) spectrometer operating at 700.13 MHz equipped with a three-channel inverse detection cyto-probe. The temperature of all measurements was 298 K. All the spectra for each sample were collected using the pulse program “cpmgpr1d” with a spectral width of 12 ppm, time domain (TD) 65536, D1 (4.0 sec); D20 (0.00030 sec); L4 (126), a relaxation delay of 4 s with an acquisition time of 3.12 s and averaged for 64 scans with 16 dummy scans. The total duration of CPMG sequence was 16.17 min. The NMR signals were assigned according to the existing literature database (IMDB, http://www.lmdb.ca/, accessed on November 22, 2017) and with the support of published data for sheep [19,36].

All the ^1^H-NMR spectra were manually baseline-corrected, phase-corrected and referenced to TSP using Chenomx NMR Suite software (Version 7.1, Chenomx Inc.). The residual signals of water (δH 4.68–5.00), CDC_l3_ (δH 7.48–7.68), and CD_3_OD (δH 3.32–3.36 ppm) were subtracted from the analysis. Moreover, the intensities of ^1^H-NMR spectra were scaled to the total intensity and reduced to integrated regions of equal width (0.01 ppm) corresponding to the region of δ 0.5–δ 10.0 by using MestReNova version: 14.0.0-23239. In total, 950 variables were obtained. The binned data was normalized to the total spectral area and converted to ASCII format.

### 2.12. Statistical Analysis

The experiment followed a completely randomized design. Data obtained for fatty acid parameters were analyzed using GLM procedures of SAS software (9.4) (SAS Institute Inc., Cary, NC, USA). Means were compared using the Tukey’s Honest Significant Difference (HSD) test and the level of significance was set at *p* < 0.05. The meat quality data were analyzed using a factorial design 4 × 3 (diets × *postmortem* aging periods) employed for data (pH, drip loss, cooking loss, color and shear force values). Univariate statistical analysis of NMR data was performed using GraphPad Prism version 5.0. One-way analysis of variance (ANOVA) was applied to evaluate the significance level of differences between metabolites from different meat groups. Statistical differences were considered significant at a level of *p* ≤ 0.05. Tukey’s multiple comparison test was used to study the statistical difference between means of four meat groups. The ASCII formatted files were imported to SIMCA-P+ version 13.0 32-bits (Umetrics AB, Umeå, Sweden) and subjected to multivariate data analysis (MvDA). The data was Pareto scaled. In Pareto scaling, the square root is used as a scaling factor and each variable is divided by the square root of standard deviation [37]. Usually, Pareto scaling is applied to data with a large dynamic range [38].

## 3. Results

### 3.1. Meat Quality

Values of meat pH measured at different age periods are shown in Table 3 for each type of diet. CaS significantly affected (*p* = 0.015) pH on d 1. Significant (*p* < 0.05) reduction in muscle pH was noted throughout the aging period from 0 to 7 d in diet groups. No significant interactions between diets and the aging period for drip loss (*p* = 0.555), cooking loss (*p* = 0.711) or shear force (*p* = 0.070) were observed. Drip loss significantly (*p* < 0.05) increased throughout the aging period from 0 to 7 d. A similar trend was observed in cooking loss in all groups except for the control. CaS significantly affected (*p* = 0.009) shear force on d 1. Additionally, shear force value significantly decreased on day 7 in control (*p* = 0.008) and PFL (*p* = 0.0003) groups (Table 3).

### 3.2. Colour Coordinates of LD Muscle in Dorper Sheep

Significant differences (*p* < 0.05) in lightness (L*), yellowness (b*), b*/a*, hue angle (H) and chroma (C*) of LD muscle on *postmortem* aging day 1 were observed among the treatments with supplementation of RBF. Differently, no difference was observed on day 0 and day 7. In general, lightness (L*) increased in PFL and CON; b*/a* and hue angle (H) increased in CON, PFL and PF; as the storage day progressed from day 0 to day 7. There was significant diet x aging days interaction for yellowness (b*) while no significant diet x aging days interaction was observed in all other parameters (Table 4).

### 3.3. Fatty Acid Composition of LD Muscle in Dorper Sheep

No differences (*p* > 0.05) were observed in concentrations of C12:0, C16:0, C17:0, C18:0, C20:4n-6, C20:5n-3, C22:5n-3, C22:6n-3, CLA *cis*-9 *trans*-11 and CLA *trans*-10 *cis*-12. Conversely, the concentrations of C14:0, C15:0, C16:1n-7, C18:1n-9, C18:1 *trans*-11, C18:2n-6 and C18:3n-3 were significantly affected by supplementation of different rumen bypass fats (*p* < 0.05) across the treatments. The concentrations of the total saturated FA, total unsaturated FA, total monounsaturated FA, total polyunsaturated FA, total n-6, UFA:SFA and PUFA:SFA differed significantly among the treatments, while concentrations of total n-3 and n-6:n-3 did not (*p* > 0.05) (in response to supplementation of RBF). Supplementation of diet with prilled fat with lecithin (PFL) increased concentrations of ∑UFA, ∑MUFA, ∑PUFA, ∑n-6, n-6:n-3, UFA:SFA and PUFA:SFA while the diet without RBF (CON) decreased those concentrations (Table 5).

### 3.4. Fatty Acid Composition of the Liver in Dorper Sheep

There were no observed differences (*p* > 0.05) in the concentrations of C12:0, C14:0, C15:0, C16:0, C16:1n-7, C17:0, C18:0, C18:1n-9, C18:1 *trans* 11, CLA *cis*-9 *trans*-11, C18:2n-6, C18:3n-3, C20:4n-6, C20:5n-3 and C22:5n-3 among the treatments, while the concentrations of CLA *trans*-10 *cis*-12 and C22:6n-3 differed significantly across the treatment groups. Similarly, there were no significant differences in the concentration of total saturated FA, total unsaturated FA, total monounsaturated FA, total polyunsaturated FA, total n-3, total n-6, n-6:n-3, UFA:SFA and PUFA:SFA among the treatment groups (Table 6).

### 3.5. ^1^H NMR Analyses

#### 3.5.1. ^1^H NMR Analyses of Polar and Nonpolar Metabolites Excreted from Muscle of Dorper Sheep

**Figure 1:**
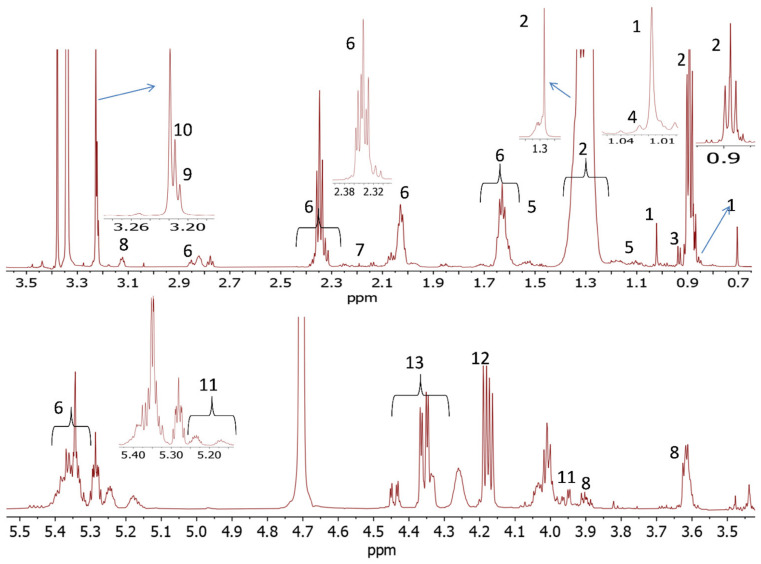
700 MHz ^1^H NMR spectrum of nonpolar extract of Dorper sheep *longissimus dorsi* muscle tissue: 1. free cholesterol, 2. sphingomyelin, 3. total cholesterol, 4. esterified cholesterol, 5. multiple cholesterol protons, 6. fatty acyl chain, 7. succinate, 8. creatine, 9. glycerophosphocholine, 10. choline, 11. glycerophospholipid backbone, 12. glycerol backbone and 13. phosphatidyl choline.

**Figure 2:**
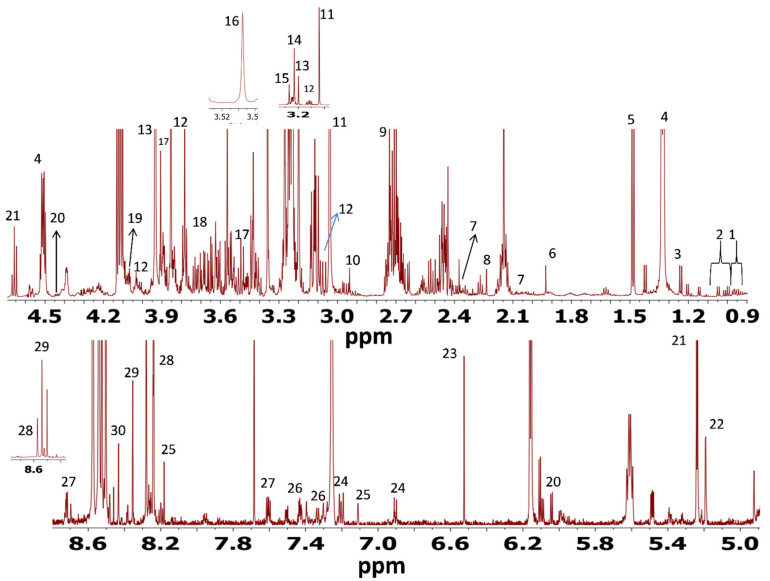
700 MHz ^1^H NMR spectrum of polar extract of Dorper sheep *longissimus dorsi* muscle tissue: 1. leucine, 2. valine, 3. 3-Hydroxybutyric aci, 4. lactate, 5. alanine, 6. acetate, 7. glutamate, 8. acetone, 9. dimethylamine, 10. trimethylamine, 11. creatine, 12. anserine, 13. glycerophosphocholine, 14. carnitine, 15. betaine, 16. glycine, 17. glutamine, 18. glycerol, 19. choline, 20. inosine, 21. glucose, 22. a-Mannose, 23. fumarate, 24. tyrosine, 25. carnosine, 26. phenylalanine, 27. niacinamide, 28. IMP, 29. ADP/AMP/ATP, and 30. formate.

A representative ^1^H NMR spectrum of nonpolar and polar metabolites in Dorper sheep *longissimus dorsi* muscle is shown in Figure 1 and Figure 2, respectively. The crowded regions of ^1^H-NMR spectrum were zoomed in to enlarge the view for better visualization. A total of 13 metabolites were identified in nonpolar (chloroform extract) fractions and 30 metabolites were identified in the methanol extract. Among the nonpolar metabolites, the signals of major metabolites corresponded to sphingomyelin, fatty acyl chain, glycerophospholipid backbone, and glycerol backbone. Among the polar metabolites, the major identified signals belonged to amino acids (alanine, glutamate, glutamine, glycine, leucine, phenylalanine, tyrosine, and valine), organic acids (3-hydroxybutyrate, acetate, creatine, formate, fumarate and lactate), nucleic acid and its derivatives (AMP/ADP, inosine), dipeptides (anserine and carnosine), vitamins (niacinamide, dimethylamine and trimethylamine), metabolites derived from amino acids (carnitine and creatine) and carbohydrates (glucose). According to signal intensities, alanine, lactate, creatine, anserine, glucose and fumarate were found to be major metabolites. The nonpolar and polar-identified metabolites, their chemical shifts and multiplicity are given in Supplementary Tables S1 and S2, respectively.

#### 3.5.2. ^1^H NMR Analyses of Polar and Nonpolar Metabolites Excreted from the Liver of Dorper Sheep

**Figure 3:**
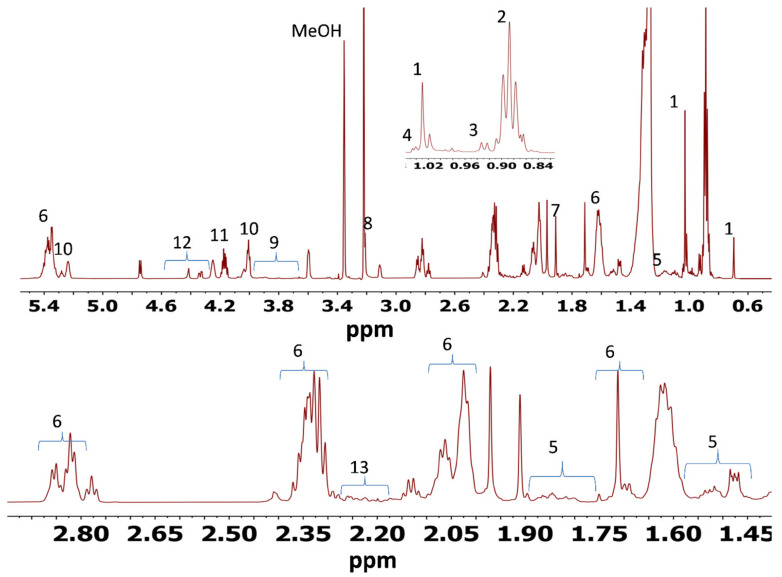
700 MHz ^1^H NMR spectrum of nonpolar extract of Dorper sheep liver tissue: 1. free cholesterol, 2. sphingomyelin, 3. total cholesterol, 4. esterified cholesterol, 5. multiple cholesterol protons, 6. fatty acyl chain, 7. acetate, 8. choline, 9. glycerophosphocholine, 10. glycerophospholipid backbone, 11. glycerol backbone, 12. phosphatidyl choline and 13. succinate.

**Figure 4:**
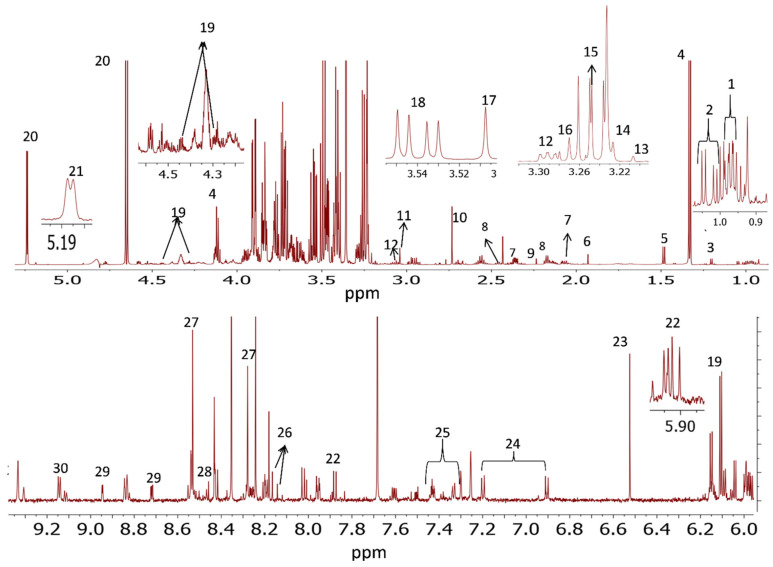
700 MHz ^1^H NMR spectrum of polar extract of Dorper sheep liver tissue: 1. leucine, 2. valine, 3. 3-Hydroxybutyric acid, 4. lactate, 5. alanine, 6. acetate, 7. glutamate, 8. glutamine, 9. acetone, 10. dimethylamine, 11. creatine, 12. anserine, 13. glycerophosphocholine, 14. choline, 15. carnitine, 16. betaine, 17. glycine, 18. glycerol, 19. inosine, 20. glucose, 21. a-Mannose, 22. uridine, 23. fumarate, 24. tyrosine, 25. phenylalanine, 26. hypoxanthine, 27. ADP/AMP/ATP, 28. formate, 29. niacinamide and 30. NADP.

A representative ^1^H NMR spectrum of nonpolar and polar metabolites in Dorper sheep *longissimus dorsi* liver is shown in Figure 3 and Figure 4, respectively. The nonpolar and polar metabolites identified using ^1^H NMR from liver tissue of Dorper sheep, their chemical shifts and multiplicity are given in Supplementary Tables S3 and S4, respectively. Like muscle tissues, a total of 13 metabolites were identified in chloroform fraction and 30 metabolites were identified in the methanol liver extract of Dorper sheep. Among the nonpolar metabolites, the signals of major metabolites corresponded to the free cholesterol and fatty acyl chains. Like muscle tissues, the ^1^H NMR spectrum of methanol extract of liver tissues showed the presence of amino acids (alanine, glutamate, glutamine, glycine, leucine, phenylalanine, tyrosine, and valine), organic acids (3-hydroxybutyrate, acetate, creatine, formate, fumarate and lactate), nucleic acid and its derivatives (AMP/ADP, inosine), dipeptides (anserine), vitamins (niacinamide, dimethylamine), metabolites derived from amino acids (carnitine and creatine) and carbohydrates (glucose). According to signal intensities, lactate, glucose and fumarate and ADP/AMP/ATP were found to be major metabolites.

#### 3.5.3. Principal Component Analysis (PCA) of NMR Data of Muscle Tissue

**Figure 5:**
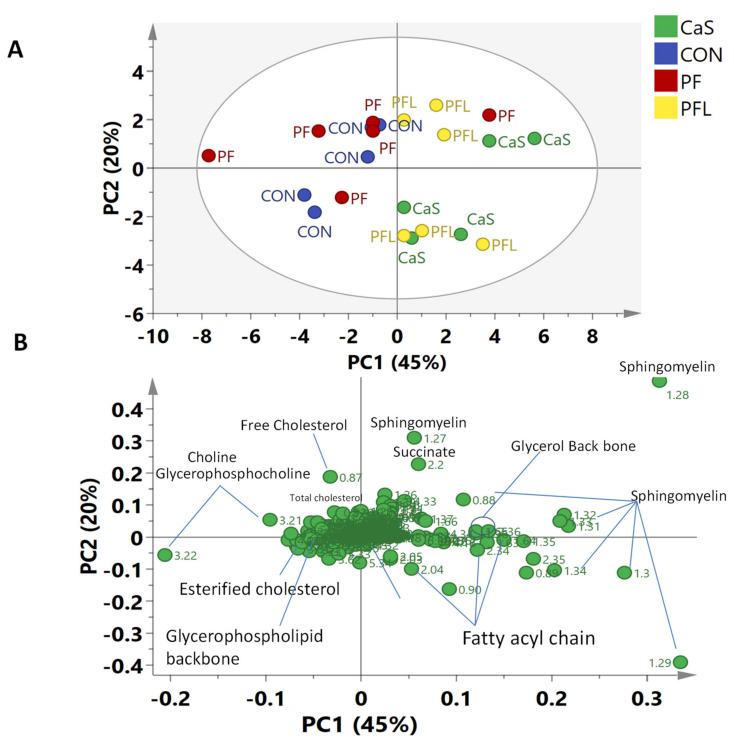
(**A**) Score plot of *longissimus dorsi* muscle of Dorper sheep. (PC1 = 45%, PC2 = 20%); CON = Basal diet without RBF, PF = Basal diet plus prilled fat, PFL = Basal diet plus prilled fat with lecithin, CaS = Basal diet plus calcium soap. (**B**) PCA Loadings scatter plot of Dorper sheep *longissimus dorsi* muscle.

In the case of muscle tissue, the PCA showed that the first four components were able to explain 87% of the variation in the data (R^2^X = 0.87, Q^2^ = 0.48). The PC1 accounted for 45% of the variation, whereas 20% variation was based on PC2. The PCA scores plot shows the projection of four treatment groups supplemented with different rumen bypass fats (Figure 5A). It was noted that the sheep fed a diet without rumen bypass fats and a diet with prilled fat (CON and PF) were clustered on the negative side of PC1 and the groups fed a diet having RBF with lecithin and calcium soap (PFL and CaS) were grouped on the positive side of PC1 (Figure 5A). However, none of the samples (from any group) were clustered tightly on any one side of PC2 and projected on either side of PC2.

**Figure 6:**
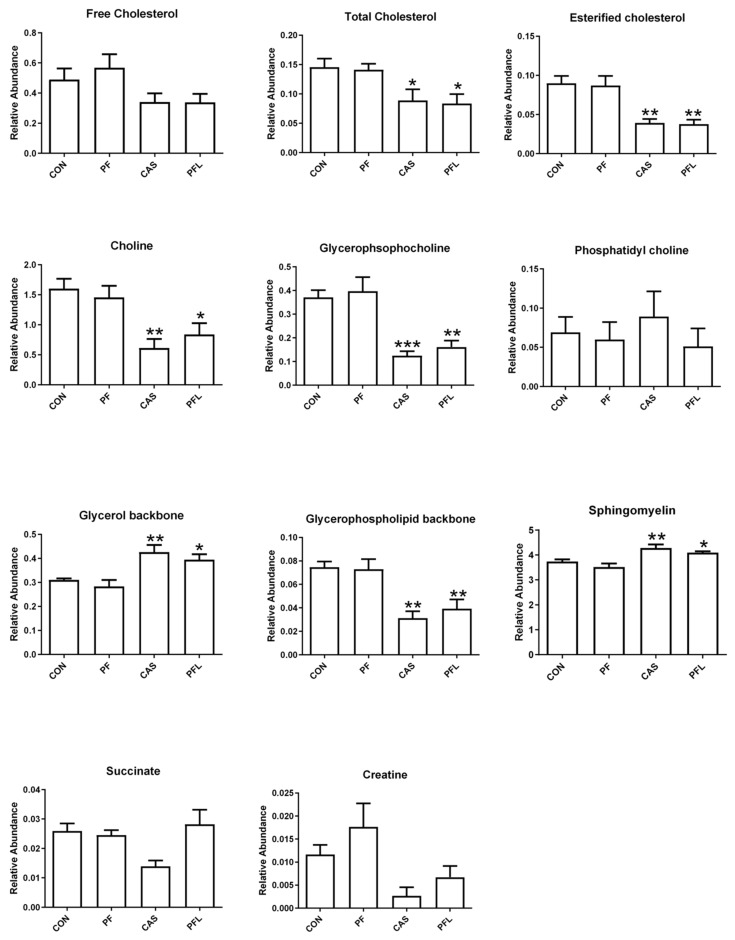
Relative quantification of the major differentiating metabolites in muscle based on the mean peak area of the related NMR signals. CON = Basal diet without RBF, PF = Basal diet plus prilled fat, CAS = Basal diet plus calcium soap, PFL = Basal diet plus prilled fat with lecithin. * depict the differences between control (CON) and the different RBF. Statistical icons: * *p* < 0.05, ** *p* < 0.01, and *** *p* < 0.001.

The corresponding loadings scatter plot showed the variables (i.e., metabolites) responsible for the separation of sheep groups in the scores plot (Figure 5B). The signals of succinate, sphingomyelin, glycerol backbone and fatty acyl chain were on the positive side of PC1, corresponding to PFL and CaS. The signals of choline, glycerophosphocholine, free cholesterol, esterified cholesterol and glycerophospholipid—identified on the negative side of PC1—were related to CON and PF. The relative quantification of identified metabolites in all four sheep groups is shown in Figure 6.

The relative quantification of the major differentiating metabolites found in the LD muscle of sheep showed that total cholesterol, esterified cholesterol, choline, glycerophosphocholine and glycerophospholipid backbone were significantly lower in CaS and PFL diets compared to CON and PF diets. Meanwhile, glycerol backbone and sphingomyelin were significantly higher in CaS and PFL compared to CON and PF diets. However, no significant differences were observed in free cholesterol, phosphatidyl choline, succinate or creatine.

#### 3.5.4. Principal Component Analysis (PCA) of NMR Data of Liver Tissue

**Figure 7:**
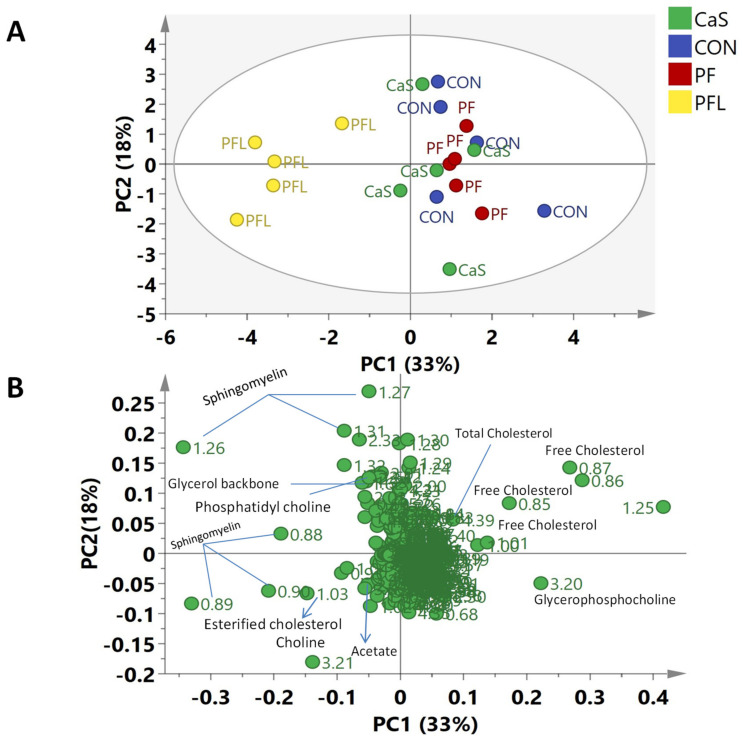
(**A**) PCA scores plot of Dorper sheep liver. (PC1 = 33%, PC2 = 18%); CON = Basal diet without RBF, PF = Basal diet plus prilled fat, PFL = Basal diet plus prilled fat with lecithin, CaS = Basal diet plus calcium soap. (**B**) PCA Loadings scatter plot of Dorper sheep *longissimus dorsi* liver.

**Figure 8:**
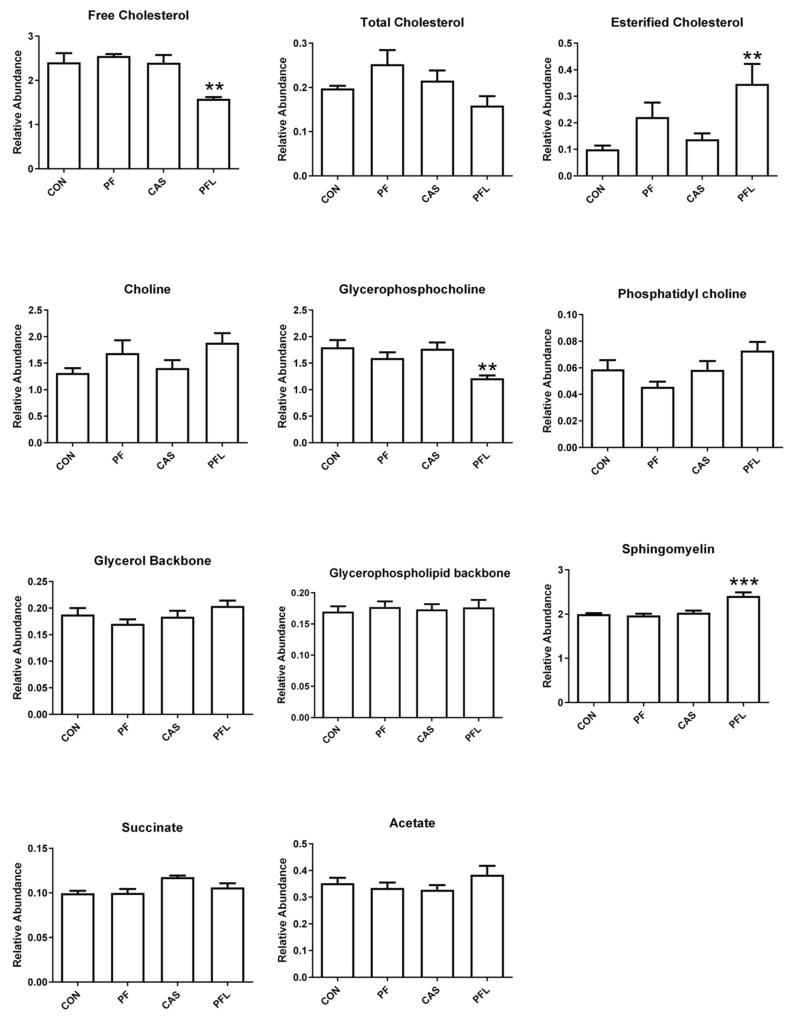
Relative quantification of the major differentiating metabolites in the liver. based on the mean peak area of the related NMR signals. CON = Basal diet without RBF, PF = Basal diet plus prilled fat, CAS = Basal diet plus calcium soap, PFL = Basal diet plus prilled fat with lecithin. Statistical icons: ** *p* < 0.01, and *** *p* < 0.001.

The ^1^H-NMR binned data of liver tissues was also subjected to MvDA to study the effects of different rumen bypass fats on the metabolites of liver tissues. The first four components were able to explain 76% of the variation in the data (R^2^X = 0.756, Q^2^ = 0.548). The PC1 accounted for 33% of the variation, whereas 18% of the variation was based on PC2. Figure 7A shows that PFL is clustered to the negative side of PC1; the rest of the groups (CaS, CON and PF) are projected to the positive side. PC3, explaining 13% of the variation, successfully separated PF (having positive PC1 and PC3 scores) from CON and CaS (placed in positive PC1 and negative PC3 quadrant) (data not shown). The corresponding loadings plot showed that the signals of total cholesterol, free cholesterol and glycerophosphocholine were found in the positive side of PC1, corresponding to CON, CaS and PF sheep groups. Sphingomyelin, choline, phosphatidyl choline, glycerol backbone and esterified cholesterol were found to be on negative PC1 quadrant, corresponding to PFL (Figure 7B). The relative quantification of identified metabolites in all four sheep groups is shown in Figure 8.

The relative quantification of the major differentiating metabolites found in the liver of sheep showed that the PFL diet induced significantly higher concentrations of esterified cholesterol and sphingomyelin and significantly lower concentrations of free cholesterol and glycerophosphocholine, compared to all three diets (CON, PF, CaS) (Figure 8). Meanwhile, total cholesterol, choline, phosphatidyl choline, glycerol backbone, glycerophospholipid backbone, succinate and acetate did not show any significant difference.

## 4. Discussion

The most important meat quality indicator is the pH value of meat [39]. Post slaughter, anaerobic metabolic decomposition of the glycogen in the muscles causes the production of lactic acid and a subsequent reduction in pH. This may lead to denaturization of muscle proteins, which ultimately results in meat with poor water-holding capacity and (in extreme cases) pale, soft and exudative (PSE) meat [40]. Regardless of diet and muscle, there was a significant decrease in pH on d 1 and d 7 postmortem. This finding was expected, as the only source of energy for muscle following the exsanguination of an animal is anaerobic glycolysis, which demands the conversion of glycogen to lactic acid in the postmortem muscle [41,42]. Glycogen is the substrate for energy production. During the first day postmortem, glycogen is converted into lactic acid and energy. The role of glycogen in skeletal muscle is thought to be influenced less by nutrition and more by the effects of stress or the energy demands of muscle [43]. The formation of lactate causes a reduction in postmortem pH [44], therefore, the decrease in pH on day 1 indicated the accumulation of lactic acid. Similar to the present study, several other researchers have reported similar pH values with supplementation of bypass fat and oils in sheep [45,46,47,48] and beef cattle [49]. In the present study, operations such as loading, transportation and handling at the slaughterhouse were carried out appropriately, without having a negative impact on animals, therefore, the pH values remained within the normal range for meat [41,50].

The water-holding capacity (WHC) is the capability of meat to retain its inherent water during storage and processing [41], which is an important factor that influences the quantity and yield of the meat [51,52] and is considered one of the important economic criteria for the meat processing industry and consumers [53]. Most of the water is held in the interfilament spaces within the myofilament lattice. The pH, sarcomere length, ionic strength, osmotic pressure and pre- or post-rigor status of the muscle are the factors which determine the volume of interfilament spaces. This, in turn, controls the amount of water present in the meat [54]. The presence of collagenase enzymes that fragment the connective tissues and myofibrillar proteins are responsible for improving the water-holding capacity of the meat [55].

The drip loss and cooking loss of the LD muscles in sheep were not influenced by the diets in the present study. The findings are supported by Bhatt et al. [56] who found similar water-holding capacities in lambs fed with RBF. Sutter et al. [45] also found similar results for WHC and cooking loss in lambs with supplementation of RBF, coconut oil and oilseeds. Similarly, no differences in WHC were found with or without RBF supplementation in cattle [57]. Similar findings were reported by several studies [46,48,58]. In contrast to the present study, Oliveira et al. [49] found a significant difference in the water-holding capacity, as the WHC was higher in linseed oil supplementation in comparison to RBF in cattle. They reported that there was no obvious explanation for this, indicating that it required further investigation. The pH of the meat can also affect other meat quality traits, including thawing loss, water-holding capacity, cooking loss and shear force [48]. Hence, similar pH in the present study yielded similar water-holding capacity and cooking loss. The aging time increased drip loss and cooking loss values in the current study, irrespective of the treatments.

Cooking loss is an accumulation of liquid and soluble matters lost from meat during cooking [41] and is important, since the remaining water in the cooked product is the main contributor to the sensation of juiciness [50]. Similar to the present study, there were no significant differences found for cooking loss with protected and unprotected lipid supplementation in cattle [49,59,60,61]. On the contrary, there was an increase in cooking loss found with RBF supplementation in lambs [56] and several other studies reported significantly different cooking loss with RBF supplementation [47,57,62,63].

Tenderness is among the most important attributes of meat quality as it affects the eating satisfaction of a consumer [50]. No significant effect of diet was observed for the shear force at day 0 and day 7 but the diet influenced shear force on day 1 postmortem in the present study. This difference on day 1 could possibly be due to differences in pH on day 1, as meat pH can affect shear force values [48]. Similar to the present study, Bhatt et al. [63] observed no significant effect of RBF supplementation on the shear force in the meat of lambs. The findings of the present study are also supported by several other studies who reported similar shear force values with protected fat supplementation [45,46,57,58,59,64]. In contrast to the present study, significant differences were observed in shear force values in cattle [61]. It was reported that sheep meat with shear force values lower than 2.27 kg/cm^2^ was classified as soft; meat with values between 2.28 and 3.63 kg/cm^2^ was classified as intermediately tender [48]. However, in the present study, the shear force values were below 2, indicating tender meat. Busboom et al. [65] reported that sheep meat fat became firmer as the levels of capric, myristic, palmitic and stearic acids increased in the diet. This could be the possible reason for tender meat in this study as our diets contained high levels of palmitic and stearic acids. The postmortem aging process significantly influenced shear force values. The reduction in shear force as the aging day progressed could be attributed to the weakening of myofibrillar structures by endogenous muscle proteinases [41]. This finding is consistent with the study conducted by Andrade et al. [57], which found a decrease as the aging day progressed with RBF supplementation when compared to the diet without RBF. The decrease in shear force during postmortem aging was also observed in mutton [66] and chevon [67]. The shear force values in the *longissimus dorsi* muscle decreased, ultimately resulting in improved tenderness with postmortem aging day from 0 to 7 in the present study.

Color is one of the most important meat quality attributes since it is the first characteristic evaluated by consumers and is an indicator of freshness. Therefore, it directly affects the final purchasing choice of the consumer [41,42]. Meat color is influenced by the animal’s age, weight, exercise, and nutrition as well as the pH of the meat. The color is most affected by the amount and chemical state of the principal pigment, myoglobin; the higher the concentration, the darker the meat [64]. The lightness (L*) and yellowness (b*) of *longissimus dorsi* muscle was not influenced by diet on postmortem aging day 0 and day 7. These were influenced, however (*p* < 0.05), on day 1, whereas, the redness (a*) was not influenced by the diet. This significant difference observed on postmortem aging day 1 was possibly due to the significant effects of diet seen in pH on day 1. The same effect was also observed in shear force values. The findings are in corroboration with Andrade et al. [57] who found a significant difference in meat lightness (L*) and redness (a*) in *longissimus lumborum* muscle with RBF supplementation in cattle. On the other hand, no significant differences were found in lightness (L*), redness (a*) or yellowness (b*) in loin muscle [49]. The lightness (L*) values in the present study are similar to the values reported by Awawdeh et al. [46] for lambs, while the redness (a*) values in the present study were less than those observed by them. Thus, the slightly darker color of the meat in the present study was due to the age of animal (mature animals were slaughtered for this work).

Ruminant products are typically high in saturated fatty acids (SFA), followed by monounsaturated fatty acids (MUFA) and polyunsaturated fatty acids (PUFA). Red meats from ruminants are higher in the ratio of saturated fatty acids (SFA) than unsaturated fatty acids (UFA) compared to that of meat products from monogastric animals and fishes [68,69]. Lipid supplementation is the main method used to modify the fatty acid profile of red meat [70,71,72]. This was clearly demonstrated in the present study, in which significant improvements in desirable fatty acids were observed in tissues of Dorper sheep with supplementation of prilled fat with lecithin.

The most abundant (37% of total fatty acids) fatty acid found in *longissimus dorsi* (LD) muscle in sheep was oleic acid (C18:1n-9), irrespective of the dietary treatment, and its concentration was influenced by the diet. The findings are in agreement with the results reported by Andrade et al. [57] who found C18:1n-9 in the most abundant concentration in rearing and fattening periods (39.75 and 41.05%) regardless of treatment with supplementation of RBF in cattle. Similarly, Oliveira et al. [49] reported C18:1n-9 ranging 37% to 40% in *longissimus thoracis* muscle with two different oils and RBF supplementation. Likewise, several other studies also found C18:1n-9 in the most abundant quantity in muscles with RBF and dietary oil supplementation [59,70,73,74]. The highest concentration of C18:1n-9 in the present study was recorded in the diet containing PFL, which could be attributed to the presence of lecithin. This finding is supported by the study conducted by Li et al. [75] who found that supplementation of soy lecithin increased the concentration of C18:1n-9 in the *longissimus dorsi* muscle of cattle compared to the control diet; however, the difference was nonsignificant.

There was no significant difference in the concentration of palmitic acid (C16:0) in muscle and liver with RBF supplementation. Similar observations were reported in the liver and foreshank muscles of sheep [72]. The concentrations of stearic acid (C18:0) in LD muscle and liver with RBF supplementation did not significantly differ, which was in agreement with the study reported by Warner et al. [4] who observed similar concentration of C18:0 with RBF supplementation in LD muscle in cattle. However, the concentration of C18:0 could not have any negative effect on the meat as, when consumed by humans, C18:0 is transformed into C18:1n-9, a fatty acid that does not carry any cardiovascular risks [76]. The findings of the present study contradict several studies who found a reduction in C18:0 with dietary oil in ruminants [77,78].

The concentration of lauric acid (C12:0) was not affected by dietary treatments in LD muscle and liver. Similar findings were observed in different tissues in cattle [78]. The concentrations of myristic acid (C14:0) and pentadecanoic acid (C15:0) in LD muscle differed significantly. This finding is in agreement with those of Gilbert et al. [59] who also reported a significant reduction with RBF supplementation, as compared to other diets in steers. Lima et al. [60] also reported similar concentrations of C12:0, C14:0 C15:0 and C17:0 with RBF supplementation in cattle.

The concentrations of linoleic acid (C18:2n-6) and linolenic acid (C18:2n-3) increased significantly in LD muscle with RBF supplementation as compared to the diet without RBF. There was no significant increase observed in the liver. Conversely, a higher concentration of C18:2n-6 was observed with RBF supplementation in cattle [49]. Our findings are in concordance with the findings of Gómez et al. [79] who found a nonsignificant effect for C18:2n-6 and a significant effect for C18:3n-3; those effects were similar in liver and LD muscles, respectively, observed in the present study. Contrary to the present study, the study conducted by Gilbert et al. [59] reported significantly higher concentration of C18-2n-6 in the control group as compared to the animals fed RBF. Additionally, the present study is supported by another study which evaluated different RBF in the muscles of cattle and found that two of the RBF increased the concentration of C18:2n-6 and C18:3n-3 compared to the control group [80]. Thus, the increase in the concentrations of C18:2n-6 and C18:3n-3 in the diet with RBF (PF, PFL and CaS) (as compared to the diet without RBF (CON)) in the *longissimus dorsi* muscle and liver could be the effect of rumen bypass fat. Inertness of RBF allowed them to pass intact through the rumen, allowing its absorption in the small intestine and subsequent deposition in the tissue [49].

No significant differences were observed in the levels of CLA *Cis*-9 *Trans*-11 or CLA *Trans*-10 *Cis*-12 in LD muscle of Dorper sheep fed RBF. The concentration of CLA *Trans*-10 *Cis*-12 in the liver increased significantly with RBF supplementation compared to the diet without RBF. This increased level of CLA is supported by Oliveira et al. [49] who found a significant increase in the concentrations of CLA with oil supplementation and attributed this increase to the presence of larger levels of C18:2n-6 in the diets which is a precursor of CLA. In the present study, the diet with calcium soap (CaS) was high in C18:2n-6—this could be the reason for significantly increased CLA *Trans*-10 *Cis*-12 concentration in the liver in the group fed with calcium soap (CaS). Moreover, our results showed no significant increase in the concentration of CLA *Cis*-9 *Trans*-11, probably because of the fatty acid composition of diet. The RBF supplemented diets (PF and PFL) were not rich in C18:2n-6 compared to the diet without RBF (CON) and CaS, and thus could not cause a significant increase in the concentration of CLA.

The n-6:n-3 ratio in the tissues examined (including the muscle and the liver) ranged from 2.32 to 3.56 with RBF supplementation in the present study. According to the Department of Health and Social Security DHSS, (1994), n-6:n-3 ratio values lower than 4.0 are desirable for the prevention of cardiovascular diseases. The values observed in the present study were even lower than these desirable values, denoting that the meat from the studied animals may be in a potentially healthier category.

In general, the supplementation of RBF significantly decreased concentrations of SFA and increased the concentrations of UFA, MUFA and PUFA, compared to the diet without RBF. These findings are supported by several studies which reported a decrease in SFA and an increase in UFA as the result of oil supplementation (whether protected or unprotected) [49,70,71,77,78]. Furthermore, RBF supplementation increased the concentrations of C18:1n-9, CLA *Trans*-10 *Cis*-12, C18-2n-6 and C18-3n-3 and reduced the n-6:n-3 ratio, which is beneficial to human health. These fatty acids reduce the levels of LDL cholesterol in the blood, freeing Dorper sheep meat fed with RBF from negative effects.

The NMR-based metabolomics proved to be an effective technique for examination of the responses to dietary treatments in muscle and liver tissues of sheep. A simple 1D spectrum enabled preliminary pattern recognition analysis and identification of metabolites. In the present study, NMR also met other significant benefits for metabolomics studies, e.g., simple sample preparation, quick spectra acquisition and the nondestructive nature of the method, allowing samples to be used with other techniques, if necessary.

There have been only a few reported studies on the effects of dietary treatments on muscle and liver tissue metabolites. Palma et al. [36] conducted similar studies on the effects of nutritional treatments on the muscle and liver metabolites in three different breeds of sheep (Merino, Damara and Dorper sheep). On the basis of multivariate data analysis (MvDA), they did not find a clear separation between treatment groups for either muscles or liver; however, they observed some differences in muscle samples among breeds. Furthermore, they were unable to find differences—especially in the Dorper breed—between the two nutritional treatment groups with good quality parameters in liver and muscle tissues. On the other hand, a clear separation was observed in the present study in the metabolomes of Dorper sheep muscle tissues supplemented with different rumen bypass fats.

The key discriminating metabolites identified in muscle tissues were choline, creatine, esterified cholesterol, fatty acyl chain, free cholesterol, glycerol backbone, glycerophospholipid backbone, glycerophosphocholine, multiple cholesterol protons, phosphatidyl choline, sphingomyelin, succinate and total cholesterol. The PFL and CaS had higher levels of the glycerol, sphingomyelin, succinate, and fatty acyl chain, whereas CON and PF were characterized by high contents of free cholesterol, esterified cholesterol, total cholesterol, choline, creatine, glycerophosphocholine and glycerophospholipid backbone.

Creatine has been known to be involved in energy production in muscle tissues. Increased levels of creatine in the present study in PFL and CaS groups were reported. As reported by Jeong et al. [81] increased creatine level in muscles may delay postmortem lactate formation and decreases in pH, potentially improving the water-holding capacity. Similarly, increased levels of creatine in muscles of feed-restricted sheep groups was reported to generate energy by promoting gluconeogenesis and glycogenolysis [36]. Likewise, higher concentrations of glycerophosphocholine in feed-restricted sheep were also reported to be involved in some regulatory processes and to play an important role in muscle control (as the metabolite is the storage form of choline and the source of a methyl group) [82]. Similar to these results, an increasing trend was observed in the levels of creatine and glycerophosphocholine in CON and PF groups in this experiment.

The PFL and CaS diets significantly decreased the concentrations of free cholesterol, total cholesterol and esterified cholesterol in muscle tissues compared with CON and PF diets. The research showed that a diet consisting mainly of red meat is rich in total fats, saturated fats and cholesterol and leads to concerns regarding the risk of coronary heart disease and atherosclerosis linked with high dietary levels of lipid and cholesterol [83]. Therefore, a reduction in cholesterol levels in PFL and CaS diets was supported by the reduction in UFA in these diets in the present study. Cholesterol comes from food, then enters the digestive tract and the small intestine to be absorbed by enterocytes of the small intestine mucosa. Next, they undergo esterification into cholesterol esters. After that, lipoprotein cholesterol esters form chylomicrons, then get into the flow of lymph and end up in the bloodstream [84]. In the present study, the inclusion of bypass fat decreased the meat cholesterol and fat content of sheep meat, when compared to the control group. The amount of lipid in meat can vary extensively, depending on many factors, e.g. the animal species, the diet eaten by the animal, the degree of trimming of fat from the muscle during the various handling phases, the particular cut of meat and the cooking or processing techniques used [85]. In the present study, low levels of cholesterol in PFL and CaS diets suggested that the meat from the Dorper sheep fed with diets with PFL and CaS had lower cholesterol and were ultimately safer and healthier for consumption by humans.

Contrary to muscle tissues, no discriminating effect of diet on the metabolome of liver tissues was found. Dorper sheep have been reported to resist dehydration and quickly recover water weight loss as soon as water is available after dehydration [86]. Likewise, different feeding systems (including extensive, semiextensive and intensive systems) were unable to affect the growth performance of Dorper sheep and also affirmed to have very little effect on meat quality parameters [87]. Compared to Merino and Damara sheep, Dorper sheep showed few changes in muscle and liver metabolomes [36].

## 5. Conclusions

On the basis of the findings of the present study, it was concluded that the supplementation of different types of bypass fat in Dorper sheep did not affect meat quality parameters, including drip loss, cooking loss, and shear force in *longissimus dorsi* muscle. However, meat pH was significantly decreased. Though the RBF supplementation did not have a negative impact on meat quality, the diet containing RBF with lecithin had the ability to modify fatty acid profiles, increasing the concentration of unsaturated fatty acids and decreasing saturated fatty acids. A possible adaptation of fatty acid and cholesterol metabolites should also be considered, as fatty acid showed some variation related to increased unsaturated fatty acid composition. NMR-based metabolomics revealed that the diets containing RBF with lecithin and calcium soap had a significant impact on metabolome and meat quality compared to the other diets. Additionally, cholesterol-reducing effects were observed. NMR techniques used in this study could be employed in food fraud laboratories to identify specific markers in meat samples suspected of adulteration where other species’ meat has been substituted with sheep meat.

## Figures and Tables

**Table 1:** Fatty acid profile of rumen bypass fats (RBF).

RBF	Fatty Acids (% of Total FA)										
	C15:0	C16:0	C16:1n-9	C18:0	C18:1n-9	C18:2n-6	C18:3n-3	Σ SFA	Σ MUFA	Σ PUFA	n-6:n-3
PF	1.39 ± 0.27	72.98 ± 1.25	0.16 ± 0.30	5.16 ± 0.48	16.34 ± 0.46	3.40 ± 0.28	0.57 ± 0.37	79.53 ± 0.93	16.5 ± 0.67	3.97 ± 0.85	5.96 ± 0.81
PFL	1.15 ± 0.19	76.72 ± 0.69	0.05 ± 0.19	4.92 ± 0.48	12.85 ± 0.79	3.94 ± 0.31	0.37 ± 0.39	82.79 ± 0.86	12.90 ± 0.73	4.31 ± 0.87	10.65 ± 0.70
CaS	1.43 ± 0.15	48.31 ± 1.07	0.81 ± 0.29	4.33 ± 0.49	41.15 ± 0.43	1.64 ± 0.40	2.33 ± 0.27	54.07 ± 1.37	41.95 ± 0.37	3.97 ± 0.62	0.70 ± 0.15

Values are expressed as mean ± standard deviation. ΣSFA = Total saturated fatty acid, ΣMUFA = Total monounsaturated fatty acid, ΣPUFA = Total polyunsaturated fatty acid; PF = Prilled fat; PFL = Prilled fat with lecithin; CaS = Calcium soap of palm fatty acids.

**Table 2:** Ingredients, chemical composition and fatty acid profile of diets.

	Diets			
Ingredients (%)	CON	PF	PFL	CaS
Soybean meal	26	27	27	27
Corn starch	39	31	30	29
Palm oil	4	2	2	2
Calcium carbonate	1	1	1	1
Vitamin-premix	0.5	0.5	0.5	0.5
NaCl	0.5	0.5	0.5	0.5
Prilled fat (RBFA)	-	5	-	-
Prilled fat with lecithin (RBFB)	-	-	5	-
Calcium soap (RBFC)	-	-	-	5
Rice straw (urea treated)	29	33	34	35
Total	100	100	100	100
**Chemical composition (% DM)**				
Dry Matter (DM)	91.54 ± 0.98	92.08 ± 0.07	92.31 ± 0.23	91.95 ± 0.29
Organic Matter (OM)	93.29 ± 0.32	91.23 ± 1.82	91.31 ± 0.27	91.96 ± 0.54
Crude Protein (CP)	18.88 ± 0.49	18.57 ± 1.13	19.92 ± 3.16	19.23 ± 2.0
Ether Extract (EE)	4.98 ± 0.16	8.27 ± 0.41	8.04 ± 0.59	8.38 ± 0.40
Acid Detergent Lignin (ADL)	6.65 ± 1.32	13.33 ± 1.50	10.35 ± 3.85	4.92 ± 1.35
Acid Detergent Fibre (ADF)	17.89 ± 2.37	21.80 ± 0.94	24.33 ± 0.37	23.94 ± 2.17
Neutral Detergent Fibre (NDF)	56.06 ± 2.29	58.78 ± 2.73	60.22 ± 3.17	51.32 ± 2.62
Crude Fibre (CF)	12.67 ± 1.60	28.42 ± 3.07	30.09 ± 2.26	13.77 ± 2.60
ME (MJ/kg DM)	11.68 ± 0.20	11.65 ± 0.39	11.66 ± 0.18	11.68 ± 0.24
**Fatty acids (% of total FA)**				
C15:0	0.78 ± 0.01	1.12 ± 0.03	0.95 ± 0.15	0.60 ± 0.19
C16:0	32.93 ± 0.08	59.52 ± 1.10	61.49 ± 0.92	21.21 ± 0.77
C16:1n-9	0.20 ± 0.01	0.13 ± 0.01	0.11 ± 0.01	0.30 ± 0.09
C18:0	3.78 ± 0.09	4.87 ± 0.04	4.58 ± 0.01	2.62 ± 0.15
C18:1n-9	42.38 ± 0.09	24.20 ± 0.53	21.83 ± 0.60	52.70 ± 0.75
C18:2n-6	18.93 ± 0.10	9.69 ± 0.61	10.44 ± 0.32	21.51 ± 0.18
C18:3n-3	1.00 ± 0.11	0.47 ± 0.03	0.60 ± 0.02	1.06 ± 0.09
ΣSFA	37.49 ± 0.09	65.51 ± 1.17	67.03 ± 0.91	24.43 ± 0.93
Total Mono UFA	42.58 ± 0.10	24.33 ± 0.53	21.94 ± 0.59	53.00 ± 0.75
Total Poly UFA	19.93 ± 0.10	10.16 ± 0.65	11.04 ± 0.33	22.56 ± 0.18
n-6:n-3	18.93 ± 2.17	20.62 ± 0.02	17.40 ± 0.07	20.29 ± 0.21

Values are expressed as mean ± standard deviation. CON = Basal diet without RBF; PF = Basal diet + prilled fat; PFL = Basal diet + prilled fat with lecithin; CaS = Basal diet + calcium soap; RBFA = Rumen bypass fat-A (prilled fat); RBFB = Rumen bypass fat-B (prilled fat with lecithin); RBFC = Rumen bypass fat-C (calcium soap); ME = Metabolizable energy; ΣSFA =Total saturated fatty acid.

**Table 3:** Physical characteristics of *longissimus dorsi* muscle of Dorper sheep at different aging days.

		Treatments				*p* Value	
Parameter	Aging Day	CON	PF	PFL	CaS	Diet	Diet × Day
pH	0	5.87 ± 0.04 ^x^	5.88 ± 0.03 ^x^	5.89 ± 0.08 ^x^	5.84 ± 0.04 ^y^	0.273	0.012
	1	5.80 ± 0.03 ^by^	5.80 ± 0.03 ^by^	5.81 ± 0.02 ^by^	5.87 ± 0.08 ^ay^	0.015	
	7	5.72 ± 0.10 ^z^	5.67 ± 0.04 ^z^	5.71 ± 0.03 ^z^	5.67 ± 0.04 ^x^	0.286	
	*p* value	0.001	<0.0001	<0.0001	<0.0001		
Drip loss (%)	1	1.75 ± 0.57 ^y^	2.30 ± 0.81 ^y^	1.65 ± 0.50 ^y^	1.56 ± 1.04 ^y^	0.282	0.555
	7	2.82 ± 0.73 ^x^	3.52 ± 0.53 ^x^	3.50 ± 0.78 ^x^	3.25 ± 1.32 ^x^	0.445	
	*p* value	0.010	0.006	0.0002	0.021		
Cooking loss (%)	0	32.05 ± 1.05	32.56 ± 0.18 ^xy^	32.21 ± 0.03	32.40 ± 0.02 ^y^	0.982	0.711
	1	32.59 ± 1.22	33.39 ± 0.19 ^y^	34.54 ± 0.26	32.95 ± 0.22 ^y^	0.764	
	7	35.12 ± 0.33	35.19 ± 0.19 ^x^	36.67 ± 0.02	35.00 ± 0.05 ^x^	0.952	
	*p* value	0.112	0.014	0.051	0.024		
Shear force (kg)	0	1.65 ± 0.24 ^x^	1.53 ± 0.24	1.60 ± 0.59 ^x^	1.54 ± 0.13	0.145	0.070
	1	1.71 ± 1.61 ^a x^	1.63 ± 1.04 ^ab^	1.64 ± 1.14 ^ab x^	1.45 ± 1.3 ^b^	0.009	
	7	1.37 ± 0.23 ^y^	1.44 ± 0.23	1.34 ± 0.95 ^y^	1.49 ± 0.35	0.662	
	*p* value	0.008	0.083	0.0003	0.622		

Values are expressed as mean ± standard deviation. Superscripts a and b indicate significant differences along the same row; superscripts x, y, z, indicate significant differences along the same column. The *p* value of diet × day presented in last column is significantly different at (*p* < 0.05). CON = Basal diet without RBF, PF = Basal diet + prilled fat, PFL = Basal diet + prilled fat with lecithin, CaS = Basal diet + calcium soap.

**Table 4:** Color coordinates of *longissimus dorsi* muscle in Dorper sheep at different aging days.

		Treatments				*p* Value	
Parameter	Aging Day	CON	PF	PFL	CaS	Diet	Diet × Day
Lightness (L*)	0	27.64 ± 3.80 ^xy^	26.73 ± 2.27	26.99 ± 2.55 ^y^	27.57 ± 2.84	0.810	0.055
	1	25.86 ± 2.31 ^b y^	26.59 ± 2.37 ^b^	27.85 ± 2.95 ^ab y^	30.00 ± 4.51 ^a^	0.012	
	7	30.76 ± 5.23 ^x^	28.64 ± 3.86	31.24 ± 2.91 ^x^	28.93 ± 4.30	0.264	
	*p* value	0.013	0.130	0.001	0.276		
Redness (a*)	0	8.32 ± 0.93	8.45 ± 1.62	8.97 ± 1.21	8.98 ± 1.64	0.422	0.784
	1	8.05 ± 0.47	8.16 ± 0.96	8.90 ± 1.27	8.43 ± 0.81	0.080	
	7	7.95 ± 1.95	8.05 ± 1.78	8.21 ± 2.16	8.12 ± 1.47	0.957	
	*p* value	0.627	0.405	0.165	0.551		
Yellowness (b*)	0	6.27 ± 099. ^y^	5.96 ± 1.29	6.24 ± 1.34	6.36 ± 1.21	0.838	0.048
	1	5.86 ± 0.71 ^c y^	6.20 ± 0.77 ^bc^	6.86 ± 1.20 ^ab^	7.37 ± 0.91 ^a^	0.0003	
	7	7.39 ± 1.04 ^x^	6.44 ± 0.89	7.21 ± 1.74	6.77 ± 1.41	0.222	
	*p* value	0.0003	0.460	0.209	0.091		
b*/a*	0	0.75 ± 0.09 ^y^	0.80 ± 0.05 ^xy^	0.78 ± 0.09 ^y^	0.80 ± 0.07	0.286	0.114
	1	0.76 ± 0.09 ^c y^	0.86 ± 0.07 ^b y^	0.77 ± 0.09 ^c y^	0.89 ± 0.17 ^a^	0.003	
	7	1.08 ± 0.65 ^x^	0.90 ± 0.23 ^x^	0.95 ± 0.16 ^x^	0.87 ± 0.20	0.466	
	*p* value	0.004	0.049	0.0003	0.341		
Hue angle (H)	0	36.85 ± 3.35 ^y^	38.67 ± 1.74	37.75 ± 3.35 ^y^	38.68 ± 2.46	0.268	0.065
	1	36.90 ± 3.36 ^ab y^	39.25 ± 2.62 ^b^	38.50 ± 3.11 ^ab y^	40.12 ± 5.06 ^a^	0.004	
	7	43.75 ± 5.64 ^x^	41.18 ± 6.58	43.10 ± 4.58 ^x^	40.38 ± 6.24	0.586	
	*p* value	0.007	0.055	0.0003	0.403		
Chroma (C*)	0	9.43 ± 0.22 ^y^	9.54 ± 0.10	10.13 ± 0.17	10.21 ± 0.15	0.591	0.402
	1	9.97 ± 0.22 ^c xy^	10.26 ± 0.16 ^b^	10.25 ± 0.17 ^a^	11.24 ± 0.25 ^a^	0.004	
	7	10.95 ± 0.41 ^x^	10.97 ± 0.32	10.66 ± 0.23	11.47 ± 0.31	0.566	
	*p* value	0.045	0.529	0.363	0.209		

Values are expressed as mean ± standard deviation. Superscripts a, b, c, indicate significant differences along the same row; superscripts x and y indicate significant differences along the same column. CON = Basal diet without RBF, PF = Basal diet + prilled fat, PFL = Basal diet + prilled fat with lecithin, CaS = Basal diet + calcium soap; L*, Measure of darkness to lightness (a greater value indicates a lighter colour); a*, The greater value indicates redder colour; b*, The greater value indicates more yellow color; C*, Chroma or saturation index is a measure of total color/vividness of color (greater value indicates greater total color/more vivid color) C* = √*a*^2^ × *b*^2^; Hue, Hue angle = tan − 1 (*b*/*a*) × 180/π.

**Table 5:** The fatty acid composition of *longissimus dorsi* muscle in Dorper sheep.

	Treatments					
Parameter	CON	PF	PFL	CaS	SEM	*p* Value
C12:0	1.32 ± 0.26	1.10 ± 0.24	0.94 ± 0.18	1.08 ± 0.35	0.054	0.080
C14:0	1.70 ± 0.28 ^a^	1.44 ± 0.31 ^ab^	1.20 ± 0.25 ^b^	1.48 ± 0.33 ^ab^	0.063	0.035
C15:0	1.62 ± 0.19 ^a^	1.36 ± 0.32 ^ab^	1.12 ± 0.10 ^b^	1.28 ± 0.46 ^ab^	0.064	0.036
C16:0	20.40 ± 0.59	19.64 ± 0.54	19.53 ± 0.51	19.97 ± 0.80	0.128	0.062
C16:1n-7	0.67 ± 0.19 ^b^	0.93 ± 0.19 ^b^	1.26 ± 0.23 ^a^	0.90 ± 0.31 ^b^	0.058	0.001
C17:0	2.11 ± 0.43	1.98 ± 0.31	1.82 ± 0.38	1.86 ± 0.36	0.070	0.485
C18:0	23.09 ± 0.60	23.21 ± 0.61	23.23 ± 0.51	23.25 ± 0.78	0.113	0.962
C18:1n-9	36.09 ± 0.53 ^b^	36.62 ± 0.81 ^ab^	37.16 ± 0.50 ^a^	36.36 ± 0.62 ^b^	0.135	0.026
C18:1 *trans*-11	1.87 ± 0.30 ^b^	2.01 ± 0.28 ^b^	2.35 ± 0.19 ^a^	2.05 ± 0.38 ^ab^	0.063	0.039
CLA *Cis*-9 *Trans*-11	0.99 ± 0.25	0.84 ± 0.25	0.93 ± 0.27	0.92 ± 0.19	0.045	0.723
CLA *Trans*-10 *Cis*-12	0.62 ± 0.14	0.68 ± 0.21	0.70 ± 0.12	0.72 ± 0.17	0.030	0.646
C18:2n-6	5.03 ± 0.29 ^b^	5.15 ± 0.26 ^b^	5.71 ± 0.40 ^a^	5.14 ± 0.47 ^b^	0.082	0.008
C18:3n-3	0.60 ± 0.16 ^b^	0.71 ± 0.11 ^ab^	0.84 ± 0.10 ^a^	0.75 ± 0.14 ^ab^	0.028	0.019
C20:4n-6	2.07 ± 0.41	2.24 ± 0.30	2.29 ± 0.49	2.21 ± 0.26	0.069	0.711
C20:5n-3	0.32 ± 0.12	0.34 ± 0.15	0.26 ± 0.15	0.29 ± 0.11	0.024	0.689
C22:5n-3	0.36 ± 0.24	0.58 ± 0.29	0.47 ± 0.29	0.57 ± 0.25	0.051	0.400
C22:6n-3	1.15 ± 0.13	1.17 ± 0.25	1.19 ± 0.25	1.17 ± 0.34	0.045	0.986
**Sums and ratios**						
∑SFA	50.24 ± 0.41 ^a^	48.72 ± 0.62 ^b^	47.84 ± 0.62 ^b^	48.91 ± 1.63 ^b^	0.236	0.001
∑UFA	49.77 ± 0.98 ^c^	51.28 ± 0.45 ^b^	53.16 ± 0.83 ^a^	51.08 ± 1.50 ^b^	0.295	<0.0001
∑MUFA	38.64 ± 0.69 ^c^	39.56 ± 0.68 ^b^	40.77 ± 0.41 ^a^	39.32 ± 0.68 ^bc^	0.186	<0.0001
∑PUFA	11.13 ± 0.65 ^b^	11.72 ± 0.39 ^ab^	12.39 ± 0.79 ^a^	11.77 ± 0.90 ^ab^	0.153	0.024
∑n-3	2.43 ± 0.36	2.80 ± 0.44	2.77 ± 0.57	2.77 ± 0.54	0.092	0.431
∑n-6	7.09 ± 0.43 ^b^	7.39 ± 0.36 ^b^	8.00 ± 0.58 ^a^	7.36 ± 0.57 ^b^	0.108	0.016
n-6:n-3	2.98 ± 0.48	2.70 ± 0.52	3.01 ± 0.72	2.74 ± 0.57	0.107	0.668
UFA:SFA	0.99 ± 0.02 ^c^	1.05 ± 0.02 ^b^	1.11 ± 0.02 ^a^	1.05 ± 0.05 ^b^	0.010	<0.0001
PUFA:SFA	0.22 ± 0.02 ^c^	0.24 ± 0.01 ^b^	0.26 ± 0.01 ^a^	0.24 ± 0.02 ^b^	0.004	0.001

Values are expressed as mean ± standard deviation. Superscripts a, b, c indicate significant differences along the same row; CON = Basal diet without RBF, PF = Basal diet plus prilled fat, PFL = Basal diet plus prilled fat with lecithin, CaS = Basal diet plus calcium soap, SEM = standard error of means; ∑SFA: Saturated fatty acids = (C12:0 + C14:0 + C15:0 + C16:0 + C17:0 + C18:0); ∑UFA: Unsaturated fatty acids = (∑MUFA + ∑PUFA); ∑MUFA: Monounsaturated fatty acids = (C16:1+ C18:1+ C18:1 *trans*-11); ∑PUFA: Polyunsaturated fatty acids = (C18:1 *trans*-11+ CLA *cis*-9 *trans*-11+ CLA *cis*-12 *trans*-10 + ∑n-3 + ∑n-6); ^7^∑ *n*-3: Omega-3 fatty acid = (C18:3n-3 + C20:5n-3 + C22:5n-3 + C22:6n-3); ^8^∑ *n*-6: Omega-6 = (C18:2n-6 + C20:4n-6); ^9^*n*-6/n-3= (∑ *n*-6/∑ n-3), UFA:SFA= (∑UFA)/ ∑SFA), PUFA:SFA = (∑PUFA/∑SFA).

**Table 6:** The fatty acid composition of the liver in Dorper sheep.

	Treatments					
Parameter	CON	PF	PFL	CaS	SEM	*p* Value
C12:0	0.52 ± 0.37	0.38 ± 0.29	0.36 ± 0.20	0.47 ± 0.35	0.056	0.744
C14:0	0.83 ± 0.12	0.81 ± 0.14	0.76 ± 0.18	0.75 ± 0.15	0.027	0.723
C15:0	0.66 ± 0.41	0.38 ± 0.21	0.46 ± 0.24	0.49 ± 0.35	0.059	0.394
C16:0	15.98 ± 0.99	15.53 ± 1.13	15.75 ± 0.47	15.31 ± 0.64	0.159	0.502
C16:1n-7	1.14 ± 0.34	1.07 ± 0.37	1.02 ± 0.28	1.09 ± 0.25	0.056	0.914
C17:0	2.23 ± 0.31	2.09 ± 0.29	2.19 ± 0.34	2.10 ± 0.43	0.062	0.838
C18:0	30.18 ± 0.56	29.82 ± 0.53	30.06 ± 0.29	30.17 ± 0.86	0.110	0.660
C18:1n-9	22.63 ± 0.59	22.84 ± 0.93	23.13 ± 0.70	22.66 ± 0.83	0.144	0.613
C18:1 *trans*-11	2.61 ± 0.57	2.90 ± 0.33	2.95 ± 0.27	3.10 ± 0.67	0.093	0.324
CLA *Cis*-9 *Trans*-11	1.08 ± 0.45	1.10 ± 0.25	1.01 ± 0.33	1.14 ± 0.22	0.058	0.890
CLA *Trans*-10 *Cis*-12	0.62 ± 0.50 ^b^	1.18 ± 0.31 ^a^	1.19 ± 0.29 ^a^	1.37 ± 0.25 ^a^	0.083	0.003
C18:2n-6	9.00 ± 0.33	9.15 ± 0.39	9.26 ± 0.48	9.16 ± 0.35	0.072	0.675
C18:3n-3	0.79 ± 0.09	0.80 ± 0.13	0.82 ± 0.15	0.83 ± 0.14	0.023	0.941
C20:4n-6	6.04 ± 0.33	6.31 ± 0.18	6.10 ± 0.46	6.01 ± 0.26	0.062	0.316
C20:5n-3	3.29 ± 0.30	3.31 ± 0.65	3.40 ± 0.39	3.07 ± 0.35	0.082	0.546
C22:5n-3	0.94 ± 0.17	1.05 ± 0.26	0.85 ± 0.26	1.06 ± 0.30	0.048	0.385
C22:6n-3	1.48 ± 0.18 ^a^	1.29 ± 0.25 ^a^	0.68 ± 0.24 ^b^	1.24 ± 0.24 ^a^	0.070	<0.0001
**Sums and ratios**						
∑SFA	50.39 ± 1.55	49.00 ± 1.24	49.58 ± 0.74	49.28 ± 1.76	0.265	0.291
∑UFA	49.61 ± 1.42	50.99 ± 1.67	50.42 ± 1.77	50.71 ± 1.16	0.289	0.383
∑MUFA	26.38 ± 1.13	26.81 ± 1.01	27.10 ± 0.70	26.85 ± 1.22	0.190	0.631
∑PUFA	23.23 ± 0.66	24.19 ± 0.96	23.32 ± 1.37	23.87 ± 0.61	0.185	0.213
∑n-3	6.50 ± 0.36	6.46 ± 0.83	5.76 ± 0.71	6.20 ± 0.57	0.128	0.152
∑n-6	15.04 ± 0.47	15.46 ± 0.48	15.36 ± 0.69	15.17 ± 0.55	0.103	0.494
n-6:n-3	2.32 ± 0.12 ^b^	2.43 ± 0.34 ^ab^	2.70 ± 0.33 ^a^	2.47 ± 0.32 ^ab^	0.058	0.129
UFA:SFA	0.99 ± 0.05 ^b^	1.04 ± 0.05 ^a^	1.02 ± 0.02 ^ab^	1.03 ± 0.04 ^ab^	0.009	0.138
PUFA:SFA	0.46 ± 0.02 ^b^	0.49 ± 0.03 ^a^	0.47 ± 0.02 ^ab^	0.48 ± 0.02 ^ab^	0.005	0.065

Values are expressed as mean ± standard deviation. Superscripts a and b indicate significant differences along the same row; CON = Basal diet without RBF, PF = Basal diet + prilled fat, PFL = Basal diet + prilled fat with lecithin, CaS = Basal diet + calcium soap, SEM = standard error of means; ∑SFA: Saturated fatty acids = (C12:0 + C14:0 + C15:0 + C16:0 + C17:0 + C18:0); ∑UFA: Unsaturated fatty acids = (∑MUFA + ∑PUFA); ∑MUFA: Monounsaturated fatty acids = (C16:1 + C18:1 + C18:1 *trans*-11); ∑PUFA: Polyunsaturated fatty acids = (C18:1 *trans*-11 + CLA *cis*-9 *trans*-11 + CLA *cis*-12 *trans*-10 + ∑n-3 + ∑n-6); ∑ n-3: Omega-3 fatty acid = (C18:3n-3 + C20:5n-3 + C22:5n-3 + C22:6n-3); ∑ n-6: Omega-6 = (C18:2n-6 + C20:4n-6); *n*-6/n-3= (∑ *n*-6/∑ n-3), UFA:SFA= (∑UFA)/ ∑SFA), PUFA:SFA = (∑PUFA/∑SFA).

## Data Availability

Data presented in this study are available on request from the corresponding author.

## Supplementary Materials

The following are available online at https://www.mdpi.com/article/10.3390/foods10051133/s1: the tables showing chemical shifts and multiplicity of identified polar and nonpolar metabolites in the muscle and liver tissue are attached as Tables S1–S4. The protocol for extraction of polar and lipophilic metabolites is attached as Supplementary Materials File S1.
