# Supplementary material for: Meat Quality, Fatty Acid Content and NMR Metabolic Profile of Dorper Sheep Supplemented with Bypass Fats
Source: Foods. 2021 May 19;10(5):1133. doi: 10.3390/foods10051133 (PMC8160904; doi:10.3390/foods10051133)
Supplement: Supplementary file 1 [file foods-10-01133-s001.zip › foods-1194645-supplementary.pdf]

foods

Table S1. ^{1}H NMR chemical shifts and multiplicity of identified non-polar metabolites in the muscle of Dorper sheep.

Metabolites	Chemical shift and Multiplicity
Choline	3.21 (s), 4.06 (dd)
Creatine	3.04 (s), 3.93 (s)
Esterified cholesterol	(1.02, s)
Fatty acyl chain	1.55–1.65 (m), 1.98–2.09 (m), 2.24–2.35 (m), 2.77–2.87 (m), 5.29–5.43 (m)
Free cholesterol	1.01 (s), 3.48–3.57 (m), 0.68 (s), 0.86 (d)
Glycerol backbone	4.15/4.29 (m), 5.26 (m)
Glycerophospholipid back-bone	3.96 (s), 5.17–5.24 (m)
Glycerophosphocholine	3.20 (s), 3.63 (m), 3.67 (m), 3.90 (m), 4.30 (m)
Multiple cholesterol protons	1.05–1.19, 1.42–1.55; 1.79–1.88
Phosphatidyl choline	3.81 (s-broad), 4.32–4.43 (m)
Sphingomyelin	3.32 (s), 0.88 (t), 1.24–1.37 (m)
Succinate	2.20 (s)
Total cholesterol	0.91 (d)

s = singlet, d = doublet, t = triplet, q = quartet, m = multiplet

Table S2. ^{1}H NMR chemical shifts and multiplicity of identified polar metabolites in the muscle of Dorper sheep.

Metabolites	Multiplicity
Acetate	1.92 (s)
Acetone	2.22 (s)
ADP/AMP/ATP	6.11(d), 8.27(s); 8.54(s)
Alanine	1.48 (d)
a-Mannose	5.18 (d)
Anserine	3.28 (m), 3.08 (m), 3.86 (s)
Betaine	3.27 (s), 3.90 (s)
Carnosine	8.18 (s), 7.12 (s), 2.7 (m), 4.5 (m)
Carnitine	3.24 (s)
Choline	3.22 (s), 4.06 (dd)
Creatine	3.04 (s), 3.93 (s)
Dimethylamine	2.73 (s)
Formate	8.45 (s)
Fumarate	6.51 (s)
Glucose	5.24 (d), 4.65 (d)
Glutamate	2.06 (m), 2.37 (m), 3.76 (m)
Glutamine	2.12 (m), 2.44 (m), 3.70 (m)
Glycerol	3.54 (dd), 3.62 (dd)
Glycerophosphocholine	3.20 (s), 3.63 (m), 3.67 (m), 3.90 (m), 4.30 (m)
Glycine	3.51 (s)
3-Hydroxybutyric acid	1.21 (d)
Inosine	3.88 (m), 3.83 (m), 4.28 (m), 4.44 (m), 6.12 (d)
Inosine Monophosphate (IMP)	8.56 (s), 8.22 (s)
Lactate	1.34 (d), 4.13 (q)
Leucine	0.94 (d), 0.96 (d), 3.72 (t)
Niacinamide	7.60 (m), 8.71 (dd), 8.94 (m)
Phenylalanine	7.32 (m), 7.37 (m), 7.42 (m)
Trimethylamine	2.92 (s)
Tyrosine	3.06 (dd), 6.88 (d), 7.19 (d)
Valine	0.99 (d), 1.05 (d), 2.28 (m), 3.62 (d)

s = singlet, d = doublet, t = triplet, q = quartet, m = multiplet

Table S3. ^{1}H NMR chemical shifts and multiplicity of identified non-polar metabolites in the liver of Dorper sheep.

Metabolites	Chemical shift and Multiplicity
Acetate	1.90 (s)
Choline	3.21 (s), 4.06 (dd)
Esterified cholesterol	1.02 (s)
Fatty acyl chain	1.55–1.65 (m), 1.98–2.09 (m), 2.24–2.35 (m), 2.77–2.87 (m), 5.29–5.43 (m)
Free cholesterol	1.01 (s), 3.48–3.57 (m), 0.68 (s), 0.86/0.87 (d)
Glycerol backbone	4.15/4.29 (m), 5.26 (m)
Glycerophospholipid backbone	3.96 (s), 5.17–5.24 (m)
Glycerophosphocholine	3.20 (s), 3.63 (m), 3.67 (m), 3.90 (m), 4.30 (m)
Multiple cholesterol protons	1.05–1.19, 1.42–1.55; 1.79–1.88
Phosphatidyl choline	3.81 (s-broad), 4.32–4.43 (m)
Sphingomyelin	3.32 (s), 0.88 (t), 1.24–1.37 (m)
Succinate	2.20 (s)
Total cholesterol	0.91 (d)

s = singlet, d = doublet, t = triplet, q = quartet, m = multiplet

Table S4 ^{1}H NMR chemical shifts and multiplicity of identified polar metabolites in the liver of Dorper sheep.

Metabolites	Multiplicity
Acetate	1.92 (s)
Acetone	2.22 (s)
ADP/AMP/ATP	6.12(d), 8.27(s); 8.54(s)
Alanine	1.48 (d)
a-Mannose	3.94 (s), 5.18 (d)
Anserine	3.28 (m), 3.08 (m), 3.87 (s)
Betaine	3.27 (s)
Carnitine	3.24 (s)
Choline	3.22 (s), 4.06 (dd)
Creatine	3.04 (s), 3.93 (s)
Dimethylamine	2.73 (s)
Formate	8.45 (s)
Fumarate	6.51 (s)
Glucose	5.24 (d), 4.65 (d)
Glutamate	2.06 (m), 2.37 (m), 3.76 (m)
Glutamine	2.12 (m), 2.44 (m), 3.70 (m)
Glycerol	3.54 (dd), 3.62 (dd)
Glycerophosphocholine	3.20 (s), 3.63 (m), 3.67 (m), 3.90 (m), 4.30 (m)
Glycine	3.50 (s)
3-Hydroxybutyric acid	1.21 (d)
Hypoxanthine	8.15 (s), 8.19 (s)
Inosine	3.85 (m), 4.28 (m), 4.44 (m), 6.11 (d)
Lactate	1.34 (d), 4.13 (q)
Leucine	0.96 (d), 0.98 (d), 3.72 (t)
NADP	9.34 (s), 9.15 (d), 8.84 (d), 8.26 (dd)
Niacinamide	7.60 (m), 8.27 (td), 8.71 (dd), 8.96 (m)
Phenylalanine	7.32 (m), 7.37 (m), 7.42 (m)
Tyrosine	3.06 (dd), 3.20 (dd), 3.95 (dd), 6.84 (d), 7.17 (d)
Uridine	7.87 (d), 5.90 (d), 5.91 (d), 4.12 (m), 3.90 (dd)
Valine	0.99 (d), 1.05 (d), 2.28 (m), 3.62 (d)

s = singlet, d = doublet, t = triplet, q = quartet, m = multiplet

Appendix A

Combined polar and lipophilic metabolites were extracted using the protocol of Viant, (2007). Briefly, three 2 ml screw top glass vials (Agilent) were labelled for each tissue sample. Micro spoons, metal spatula, mortar and pestle were precooled using liquid nitrogen, glass vials were pre-cooled by placing them on dry ice and the solvents were kept refrigerated at 4° C. All sample handling and storage was conducted in glass because chloroform could leach compounds from plastic tubes and pipet tips, which could contaminate the NMR spectra. Frozen tissue (150 mg) was pulverized carefully with a precooled mortar and pestle using liquid nitrogen to a fine powder for 15 to 30 seconds. The tissue was kept frozen at all times, more liquid nitrogen was added whenever needed. After grinding little liquid nitrogen was added to consolidate the frozen powder in the bottom of the mortar, scrapped up and collected quickly with precooled micro spoon into a pre-weighed glass vial (sitting in dry ice). The process was repeated for all the tissue samples. The glass vials containing tissue powder was reweighed and wet tissue mass of each sample was calculated making sure to keep all samples frozen on dry ice. Then 4 mL/g methanol and 0.85 mL/g deionized water was added to the first frozen sample and vortexed for 60 seconds. Then 2 mL/g chloroform was added followed by an additional vortexing for 60 seconds and were kept on dry ice. The process was repeated for all the samples. All the samples were then mixed on the ice, using an orbital shaker at 300 rpm for 10 min. All solutions became monophasic. Then 2 mL/g chloroform and 2 mL/g deionized water was added to each sample, vortexed for 60 seconds and centrifuge at 1000*g* for 15 min at 4°C. The solutions were separated into an upper methanol:water phase (with polar metabolites) and a lower chloroform phase (with lipophilic compounds), separated by protein debris. Using two glass Pasteur pipets, the upper and the lower layers of each sample were transferred into separate small glass vials. The solvents were lyophilized and stored at - 80° C until required.
